# Effect of Low-Temperature Plasma Sterilization on the Quality of Pre-Prepared Tomato-Stewed Beef Brisket During Storage: Microorganism, Freshness, Protein Oxidation and Flavor Characteristics

**DOI:** 10.3390/foods14071106

**Published:** 2025-03-22

**Authors:** Qihan Shi, Ying Xiao, Yiming Zhou, Jinhong Wu, Xiaoli Zhou, Yanping Chen, Xiaodan Liu

**Affiliations:** 1 School of Perfume and Aroma Technology, Shanghai Institute of Technology, Shanghai 201418, China; 13916902288@163.com (Q.S.); zhouxlsit@163.com (X.Z.); 2 School of Food and Tourism, Shanghai Urban Construction Vocational College, Shanghai 201415, China; liuxiaodan@succ.edu.cn; 3 School of Agriculture and Biology, Shanghai Jiao Tong University, Shanghai 200240, China; wujinhong@sjtu.edu.cn (J.W.); catherinechenyp@163.com (Y.C.)

**Keywords:** low-temperature plasma sterilization, tomato-braised beef brisket, pre-prepared meal, oxidative stability, volatile compounds, microbial inactivation, high-temperature short-time sterilization

## Abstract

Traditional tomato-braised beef brisket with potatoes is celebrated for its rich, complex flavors and culinary appeal but requires lengthy preparation. Pre-packaged versions of the dish rely on thermal sterilization for safety; however, high-temperature processing accelerates protein and lipid oxidation, thereby compromising its sensory quality. As the demand for ready-to-eat meals grows, the food industry faces the challenge of ensuring microbial safety while preserving flavor integrity. In this study, low-temperature plasma sterilization (LTPS) (160 KV, 450 s) was evaluated as a non-thermal alternative to conventional high-temperature short-time (HSS) sterilization. Furthermore, a comprehensive analysis was conducted over a 10-day storage period, assessing microbial viability, physicochemical properties (pH, shear force, and water-holding capacity), oxidative markers (TBARS, TVB-N, and protein carbonyls), volatile compounds (GC-MS), and electronic nose (e-nose) responses. The results revealed that LTPS (160 kV, 450 s) successfully maintained bacterial counts below regulatory limits (5 lg CFU/g) for 72 h, ensuring that the microbial indicators of short-term processed products sold to supermarkets through cold chain logistics were in the safety range. Additionally, LTPS-treated samples showed a 4.2% higher water-holding capacity (*p* < 0.05) during storage, indicating improved preservation of texture. Furthermore, LTPS-treated samples exhibited 32% lower lipid oxidation (*p* < 0.05) and retained 18% higher sulfhydryl content (*p* < 0.05) compared to HSS, indicating reduced protein oxidation. GC-MS and e-nose analyses showed that LTPS preserved aldehydes and ketones associated with meaty aromas, while HSS contributed to sulfur-like off-flavors. Principal component analysis showed that the LTPS samples had shorter distances across various storage periods compared to HSS, indicating reduced differences in aroma difference. The findings of this study demonstrate LTPS’s dual efficacy in microbial control and aroma preservation. The technology presents a viable strategy for extending the shelf life of pre-prepared meat dishes while reducing oxidative and flavor deterioration, thereby establishing a solid foundation for LTPS application in the pre-prepared food sector.

## 1. Introduction

Pre-prepared meals are foods that have been pre-processed and packaged for consumption after simple heating or cooking. Although renowned for their variety and delicacy, traditional Chinese dishes involve lengthy preparation and complicated cooking processes. The revenue of the global pre-prepared meal market is projected to reach USD 701.3 billion by 2029. As of 2025, China is expected to contribute approximately USD 170 billion, accounting for about a quarter of the total market revenue [1]. The popularity and growth of pre-prepared meals has also received a significant boost with the COVID-19 pandemic. Traditional Chinese dishes, such as tomato-braised beef brisket with potatoes, are highly valued for their complex flavors and rich taste but require lengthy preparation time and intricate cooking process. In order to ensure extended shelf life, pre-prepared meat dishes undergo high-intensity sterilization, which negatively impacts the taste and nutritional value of the dishes due to the acceleration of protein and lipid oxidation. As a result, consumers are concerned about the quality of these products.

Thermal sterilization is the most commonly used method in pre-prepared dish production, including high-temperature short-time sterilization, and pasteurization [2]. While it effectively eliminates most pathogens to ensure food safety [3,4], it also promotes protein and lipid oxidation, leading to nutritional loss and flavor changes. The differences in the volatile components of tomato-stewed beef brisket between high-temperature sterilization (121 °C, 22 min) and pasteurization have been previously reported [5]. The findings indicated that both high-temperature sterilization and pasteurization significantly compromised the stability of volatile compounds in pre-prepared meals. For this reason, researchers have explored non-thermal sterilization methods in meat products, such as ultraviolet sterilization [6], ultra-high pressure sterilization [7,8] and ultrasonic sterilization [9] to achieve both quality preservation and shelf life extension.

Cold plasma (CP), a non-thermal sterilization technology, generates substantial amounts of reactive oxygen species (ROS) and reactive nitrogen species (RNS) by ionizing gas at room temperature, effectively inhibiting or killing microorganisms [10]. This technology is now widely used to control microbial growth in fresh meat [11,12,13]. Numerous studies [14,15,16,17] have demonstrated that low-temperature plasma treatment effectively eliminates *Listeria*, *Escherichia coli*, *Pseudomonas fluorescein*, *fungicides*, and other pathogenic bacteria and putrefactive bacteria in meat and meat products. Notably, the underlying variations in bactericidal efficacy across species stem from differences in the structures of cellular components [15]. Gram-negative bacteria (e.g., *E. coli*) are more sensitive to ROS-induced unsaturated fatty acid peroxidation in lipopolysaccharide membranes, while Gram-positive species (e.g., *Listeria*) require prolonged exposure for the penetration of reactive species through their thicker peptidoglycan layers [15,18,19].

Low-temperature plasma sterilization also affects food composition. Most studies indicate that low-temperature plasma treatment improves the shear force, color value, and thiobarbituric acid reactive substances (TBARS) of meat. By using low-temperature plasma to sterilize raw beef and raw chicken, Luo et al. [20] and Abdel-Naeem et al. [21] demonstrated that low-temperature plasma treatment can reduce the shear force of meat, improving meat tenderness. Low-temperature plasma sterilization at appropriate intensities alters volatile compounds in meat by generating reactive nitrogen and oxygen species that modify protein structures, influencing their ability to bind to flavor compounds. This effect preserves lipid-derived flavor precursors while reducing oxidative degradation of volatile components [22,23]. A study on low-temperature plasma treatment of bacon [14] reported significant changes in alcohols such as 3-methylbutanol and aldehydes such as decanal compared with the control group, leading to a significant improvement in sensory scores. These findings highlight the impact of low-temperature plasma sterilization technology on the quality of meat products. Thus, it was hypothesized that low-temperature plasma sterilization could enhance the tenderness and better preserve the initial aroma of pre-packaged tomato beef brisket dishes. However, to the best of the authors’ knowledge, limited research has been conducted on the flavor of pre-prepared meat dishes.

Thus, this study aimed to investigate the impact of low-temperature plasma sterilization (LTPS) on the quality of tomato-stewed beef brisket with potatoes during storage, focusing on microbial viability, physicochemical changes, protein oxidation, and flavor alterations. The findings of this study explored the feasibility of applying LTPS technology to pre-prepared meat dishes and its potential as a novel packaging technology for their preservation and also provides a scientific foundation for the commercial promotion of traditional Chinese dishes.

## 2. Materials and Methods

### 2.1. Sample Preparation

The beef brisket used was sourced from the same batch as the tenderloin, which came from the hind legs and was provided by Inner Mongolia Alshan Cattle Co., Ltd. (Inner Mongolia, China). Tomatoes, potatoes, and seasoning were purchased from the local supermarket and stored at −18 °C until cooking. The pre-washed and pre-processed brisket was stewed in a pot with tomatoes for 1.5 h. The prepared dish was then divided into three portions for different sterilization treatments. The net weight of each sample was 250 g.

### 2.2. Packaging and Sterilization

To minimize the risk of microbial contamination during storage, both unsterilized samples (US) and samples sterilized for short times at high temperatures (HSS) were vacuum-packed immediately after preparation. For low-temperature plasma-sterilized samples (LTPS), an acrylonitrile film packaging system integrated with the plasma treatment apparatus was used to preserve the efficacy of treatment during storage.

US served as the control group and were not subject to any sterilization. After cooking and cooling for 30 min, the tomato-stewed beef brisket was immediately vacuum-packed (PET plastic) and stored in a 4 °C refrigerator for subsequent testing.

HSS were prepared by vacuum-packing (PET plastic) the prepared tomato-stewed beef brisket, followed by autoclaving (YXQ-75SII, Shanghai Boxun Medical Biological Instrument Co., Ltd., Shanghai, China). The autoclave was set to 121 °C for 20 min. Once sterilization was complete and the samples had cooled to room temperature, they were quickly transferred to a refrigerator and stored at 4 °C.

LTPS were treated using a low-temperature plasma sterilizer (CPCS-MAP-I, Suzhou Yirun Food Technology Co., Ltd., Suzhou, China). First, the tomato-stewed beef brisket was packed and sealed with a propylene film using a packaging machine (WQ-018, Shanghai Weiqi Coding Equipment Co., Ltd., Shanghai, China). The packaged tomato-stewed beef brisket was placed in the cavity of the low-temperature plasma sterilization equipment (Saipu Fresh Preservation Technology (Suzhou) Co., Ltd., Suzhou, China) and treated for 150–450 s at 120 kv–160 kv. After sterilization, the samples were cooled to room temperature and promptly transferred to a refrigerator to store at 4 °C. Nine combinations of plasma treatment were used: 120 kv, 150 s; 120 kv, 300 s; 120 kv, 450 s; 140 kv, 150 s; 140 kv, 300 s; 140 kv, 450 s; 160 kv, 150 s; 160 kv, 300 s; 160 kv, 450 s.

### 2.3. Sample Collection

All samples had a net weight of 250 g. All sterilized samples were stored in a refrigerator at 4 °C for 10 days. The low-temperature plasma sterilization parameters were optimized based on TVC measurements within 72 h of treatment, and samples treated with the optimized parameters were monitored for TVC every 24 h during 10-day storage. Concurrent analyses conducted at 5-day intervals included physicochemical properties (pH, shear force, and water-holding capacity), freshness indicators (TVB-N and TBARS), protein oxidation (carbonyl content and sulfhydryl content), and flavor characteristics (electronic nose analysis and GC-MS).

### 2.4. Total Viable Count

The total viable count (TVC) was determined using the plate counting method. Under aseptic conditions, 25 g of meat samples were randomly taken from each group and minced into sterile sampling bags containing 225 mL of 0.85% sterilized saline solution. The samples were homogenized at medium speed for 2 min using a homogenizer, followed by serial 10-fold dilutions to obtain the desired dilution factors. Next, suitable dilutions were selected, and 1 mL of the diluted suspension was transferred into sterile Petri dishes. Approximately 15 mL of Plate Count Agar (PCA) medium was poured into each Petri dish and allowed to solidify. The plates were then incubated at 37 °C for 48 h to facilitate microbial growth. Microbial counts were recorded as log10 colony-forming units per gram (log10 CFU/g).

### 2.5. Shear Force, pH Value, and Water Holding Capacity

Shear force was measured using the method described by Wang [24] et al. with slight modifications. After cooking, the beef brisket was cooled to an ambient temperature and cut along the muscle fiber orientation into samples measuring 1.5 cm × 1.5 cm × 2 cm. A texture analyzer (TA-XT, Shanghai Tengba Instrument Technology Co. Ltd., Shanghai, China) equipped with a V-shaped shear blade was used to measure the shear force perpendicular to the muscle fiber direction. Six replicate measurements were conducted for each sample, and the average shear force value was calculated. Non-tenderized beef brisket was used as the control group. The instrumental parameters were set as follows: pre-test and test speeds of 10 mm/s, post-test speed of 5 mm/s, trigger force of 5 g, and a compression distance of 12 mm.

The pH value was measured following the method reported by Wang et al. with slight revisions. A 2.5 g portion of homogenized tomato-braised beef brisket was accurately weighed and blended with 45 mL of deionized water. Next, the mixture was homogenized for 30 s in a high-speed homogenizer and then filtered to obtain the supernatant. The pH of the collected supernatant was subsequently measured using a calibrated pH meter.

The water-holding capacity (WHC) of the samples was determined using the method reported by Gharibzahedi [25] et al. A measurement of 5.0 g of the beef brisket sample was accurately weighed (W_1_) and transferred into a pre-weighed 50 mL centrifuge tube. This was followed by centrifuging the sample at 3000× *g* for 15 min at 4 °C to separate the free water. After centrifugation, the supernatant was carefully decanted, and any residual moisture on the surface of the pellet was gently blotted with sterile filter paper. The tube containing the centrifuged sample was then reweighed (W_2_) to determine the amount of moisture. The WHC was then calculated using Equation (1):

(1) $$WHC\left(\%\right)=\frac{W_{2}}{W_{1}}\times 100$$

### 2.6. Thio Barbituric Acid Reactive Substances and Total Volatile Base Nitrogen

The degree of freshness was assessed by measuring the Thio Barbituric Acid Reactive Substances (TBARS) via a method reported by Xiong [26] et al. A measurement of 5 g (M) of chopped tomato-braised beef brisket was homogenized at 13,600 rpm for 30 s. Subsequently, 3 mL of 1% thiobarbituric acid (TBA) was added to the homogenate and shaken at 37 °C for 30 min. Next, 17 mL of 2.5% trichloroacetic acid (TCA) was added to the mixture and boiled for 30 min. After cooling, the mixture was filtered and the filtrate was centrifuged at 7000 rpm for 10 min to collect the supernatant. This was followed by adding 5 mL of chloroform to the supernatant and shaking the mixture before allowing phase separation. After the mixture was separated into phases, the upper layer was extracted and its absorbance was measured at 532 nm (A_532_) using a microplate reader (Infinite M200PRO, Tecan Laboratory Equipment Co., Ltd., Shanghai, China). Finally, the TBARS content of the sample was calculated using Equation (2):

(2) $$TBARS\left(mg/kg\right)=\frac{A_{532}}{M}\times 9.48$$

The total volatile basic nitrogen (TVB-N) contents of the samples were determined using the Semi-micro Kjeldahl method. A 5 g portion of the homogenized tomato-braised beef brisket was mixed with 37.5 mL of deionized water and mixed thoroughly using a vortex mixer. The mixture was left to stand for 30 min before being transferred to a distillation tube containing 0.5 g of MgO for TVB-N analysis. Subsequently, the distillation tube was loaded into a semi-automatic Kjeldahl nitrogen analyzer (QSY-QSY-1, Beijing Heng Odd Instrument Co. Ltd., Beijing, China). The distillation process was carried out for 120 s. The resulting distillate was collected in a receiving flask containing 20 mL of 20 g/L H₃BO₃ solution mixed with an indicator solution. Next, the ammonia absorbed in the boric acid solution was titrated with a 0.01 mol/L HCl solution and the sample’s TVB-N content was calculated using Equation (3):

(3) $$TVB-N\left(\frac{mg}{100g}\right)=\frac{(V_{1}-V_{2})\times c\times 14}{m}\times 100$$

where V_1_ represents the volume of a standard titration solution of hydrochloric or sulfuric acid, mL; V_2_ denotes the consumed volume of a standard titration solution of hydrochloric acid, mL; c represents the actual concentration of HCl, mol/L; and m refers to the weight of the tested sample, g.

### 2.7. Sulfhydryl Content and Carbonyl Content

The sulfhydryl content of the samples was determined by referring to a method reported by Bao [27] et al. with minor modifications. A measurement of 1 g of the homogenized tomato-braised beef brisket was mixed with 25 mL of 0.1 mol/L Tris-HCl buffer containing 5% sodium dodecyl sulfate (SDS). The mixture was homogenized at 13,600 rpm for 30 s using a high-speed homogenizer. The resulting homogenate was then incubated in a water bath at 80 °C for 30 min, followed by rapid cooling in ice. Next, the mixture was filtered through a standard filter paper to obtain the protein extract. Finally, a bicinchoninic acid (BCA) assay kit (P0012, Sinopharm Chemical Reagent Co., Ltd., Shanghai, China) was used to measure the protein concentration of the filtrate. The relationship used for the standard curve of Sulfhydryl Content is as follows:

$$y=10.122+5.877\;(R^{2}=0.9952)$$

The absorbance of the samples was recorded at 562 nm using a UV spectrophotometer (UV-3600i Plus, Shimadzu Corporation, Kyoto, Japan). To quantify sulfhydryl (-SH) groups, 0.5 mL of the filtered solution was mixed with 2 mL of 0.1 mol/L Tris-HCl buffer and 0.5 mL of 10 mmol/L 5,5′-D = dithiobis(2-nitrobenzoic acid (DTNB) solution. After incubating the reaction mixture in the dark for 30 min, the absorbance was measured at 412 nm. Equation (4) was used to calculate the sulfhydryl content:

(4) $$Sulfhydryl\;Content(nmol/mg)=\frac{(A_{412}-A_{0})\times 10^{6}\times 6}{13,600\times 1\times c}$$

where *A*_412_ is the sample’s absorbance; *A*_0_ is the Light absorption value of ultrapure water at 412 nm; 13,600 is the molar extinction coefficient, L·M^−1^·cm^−1^; and *c* is the protein concentration of the sample.

The carbonyl content of the samples was measured using the carbonyl rapid kit (Titan Technology Co., Ltd., Shanghai, China). The relationship used for the standard curve of carbonyl content is as follows:

$$y=5.266-5.551\;(R^{2}=0.997)$$

### 2.8. Volatile Compounds

A headspace solid-phase microextraction (HS-SPME) coupled with gas chromatography-mass spectrometry (GC-MS) (Agilent 7890&7820, Santa Clara, CA, USA) was used for volatile compound analysis, following the methodology reported by Shi [28] et al. with modifications. Two grams of the homogenized tomato beef brisket samples were weighed into a 20 mL headspace vial. After equilibration at 60 °C for 30 min, the samples were extracted using a 50/30 μm DVB/CAR/PDMS fiber (headspace adsorption for 40 min), followed by thermal desorption at 250 °C for 5 min in the GC injector.

GC Conditions: DB-Wax column (30 m × 0.25 mm × 0.25 μm); temperature program: initial 40 °C held for 2 min, then raised to 130 °C at 2 °C/min, increased further to 220 °C at 4 °C/min (maintained for 4 min), and finally raised to 250 °C at 10 °C/min (maintained for 5 min). The cCarrier gas was high-purity helium (99.999%) with flow rate 1.6 mL/min.

MS Conditions: electron ionization (EI) source at 70 eV, ion source temperature 230 °C, and mass scan range *m*/*z* 35–350.

Retention indices (RIs) were used for compound identification and cross-referenced with mass spectra in the NIST 2017 database. For quantitative analysis, 2-octanol (220 μg/mL) was used as the internal calibration standard, and analyte concentrations were determined based on peak area ratios relative to the standard. RI values were calculated using a homologous series of C6-C30 n-alkanes as reference markers.

The odor impact potential of aromatic components was evaluated through odor activity values (OAVs), which were calculated using Equation (5):

(5) $$OAV=\frac{C}{C_{t}}$$

where *C* represents the measured concentration of the target compound and *C_t_* shows its perceptual detection threshold. This dimensionless index quantifies the relative contribution of individual odorants to the overall sensory profiles.

### 2.9. E-Nose

The E-nose measurements were conducted following the method reported by Niu [29] et al. with minor modifications. Then, 2 g of tomato-braised beef brisket was placed in a 20 mL headspace vial and immediately sealed for analysis. Before testing, the sealed vial containing the sample was placed in a water bath at 60 °C for 30 min to allow for thermal equilibration and thermal volatilization of aroma compounds. The E-nose (PEN3, German Airsense Company, Shanghai, China) was operated using the following parameters: sensor purge time 180 s, carrier gas purge time 120 s, headspace sampling volume 1200 mL/min, gas flow rate 400 mL/min, and data acquisition duration 10 min. Each sample was analyzed in nine replicates.

The E-nose system utilized a sensor array comprising metal oxide semiconductor (MOS) sensors, each designed with distinct sensitivity profiles for volatile organic compounds (VOCs). Table 1 provides a comprehensive summary of sensor array specifications, including sensor types, target analytes, and detection ranges.

### 2.10. Statistical Analysis

All experiments were conducted in triplicate, and the results were expressed as mean values ± standard deviation ranges. Statistical comparisons of volatile and non-volatile components were performed using analysis of variance (ANOVA), with statistical significance thresholds set at *p* < 0.05. Data preprocessing and normalization were carried out using Origin 2021 software, while multivariate pattern visualization including heatmap generation was conducted in GraphPad Prism 10. The normalized datasets were subjected to chemometric analysis using two multivariate models for dimensionality reduction: principal component analysis (PCA) and orthogonal partial least squares-discriminant analysis (OPLS-DA), both performed with Simca 14.1 software. The OPLS-DA framework also allowed for the calculation of variable importance in projection (VIP) scores to identify chemically significant components.

## 3. Results and Discussion

### 3.1. Total Viable Count (TVC)

Table 2 presents the effects of different low-temperature LTPS parameters on the total bacterial counts, including the changes observed over three days post-sterilization. According to Chinese standards [30] and European Union standards [31] for cooked meat products, the maximum allowable limit for total bacterial counts in qualified products is 10^5^ CFU/g (equivalent to 5 lg (CFU/g)). Notably, the unsterilized control (US) samples approached this threshold after 72 h. However, both LTPS and HSS significantly reduced bacterial counts compared to the US group. Moreover, the bactericidal efficacy of LTPS increased significantly with increasing sterilization voltage and duration, consistent with previous studies [32]. The antimicrobial mechanism of CP primarily operates through two synergistic pathways mediated by distinct active components [33,34]. Reactive oxygen and nitrogen species (RONS) generated during CP treatment initiate oxidative damage to microbial cellular components, leading to irreversible inactivation. Simultaneously, the charged particle flux induces membrane destabilization through electrostatic interactions, with electroporation-mediated permeabilization further accelerating cell death [35]. The efficacy of the treatment is highly dependent on voltage, with suboptimal voltage conditions restricting both RONS yield and ion flux density, thereby reducing antimicrobial performance [33]. Conversely, optimizing voltage intensification within the effective range significantly enhances the generation of these bioactive agents, leading to a substantial improvement in microbial eradication rates.

**Figure 1:**
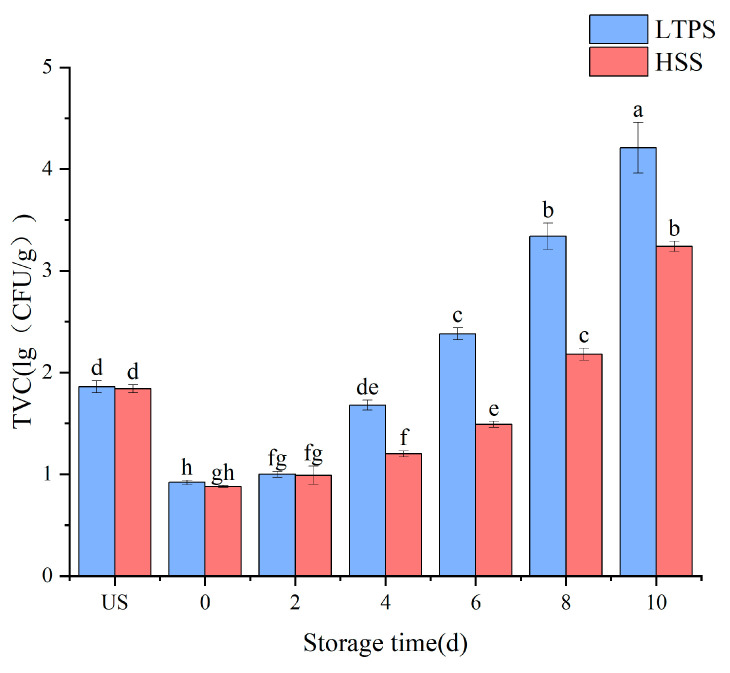
Impact of different sterilization on the TVC of tomato-stewed beef brisket. Note: different letters denote statistically significant differences (*p* < 0.05) between treatments.

For instance, sustained suppression of bacterial growth was achieved by optimizing LTPS parameters (160 kV, 450 s) and maintaining counts below 5 lg (CFU/g) over 72 h. This approach demonstrated superior antibacterial performance compared to lower-voltage or shorter-duration treatments, highlighting the critical role of voltage and exposure time in maximizing the antimicrobial potential of LTPS technology. Thus, the optimized LTPS parameters (160 kV, 450 s) were used to further the experiment during storage. Figure 1 compares the bacterial counts between HSS and LTPS over the storage period. While the TVC was higher in the LTPS samples as compared to HSS samples, all remained within the acceptable safety limit. The observed fluctuation in total viable counts during storage could be attributed to the possible resuscitation of injured microbial cells. While the optimized LTPS treatment effectively reduced the initial bacterial loads, it is possible that residual microorganisms with compromised membranes might regain metabolic activity through stress–response mechanisms, particularly when exposed to the nutrient-rich matrices of meat products [18].

### 3.2. Physicochemical Characteristics

Table 3 shows the physicochemical characteristics of tomato-stewed beef brisket during storage, including shear force, pH value, and water holding capacity. With the increase in the number of storage days, there was a significant decrease in the pH and water-holding capacity of the sample. These changes could be attributed to the microbial degradation of proteins, which compromised the structural integrity of the cells and led to a decrease in water content [36]. These results were consistent with the pH changes observed in the plasma treatment of vacuum-packed beef [37].

Tenderness is a key factor influencing consumer preference for meat, as it reflects the softness, ease of chewing, and juiciness of meat. Meat tenderness is evaluated by shear force measurements. Regardless of the sterilization method, the shear force exhibited a decreasing trend, though the reduction was not statistically significant. However, LTPS samples showed a more pronounced variation in shear force compared to the HSS samples. Previous studies have attributed this observation to the degradation of meat proteins, particularly the myofibrillar protein structure, caused by low-temperature plasma [21,38]. Moreover, high voltage generates RONS that alter the pH. These biochemical changes possibly activate calcium-dependent proteases, triggering selective degradation of myofibrillar proteins and consequent alterations in the structures of muscle fibers, influencing the shear force of the meat [33].

### 3.3. Freshness

**Figure 2:**
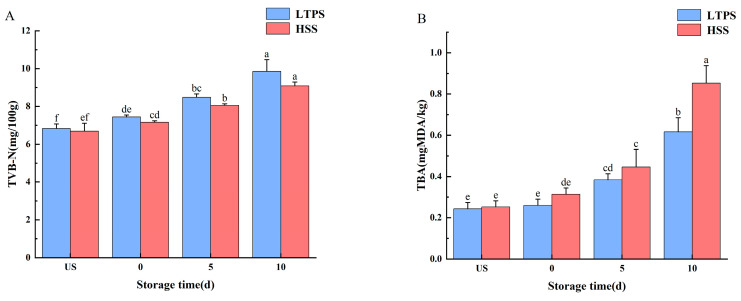
Impact of different sterilization treatments on the TBARS (**A**) and TVB-N (**B**) of tomato-stewed beef brisket during storage. Note: different letters indicate statistically significant differences (*p* < 0.05) between the treatments.

Figure 2 shows the freshness of braised beef brisket with LTPS and HSS during storage. Total volatile basic nitrogen (TVB-N) primarily includes ammonia, dimethylamine, and trimethylamine—nitrogenous compounds generated through enzymatic activity or microbial degradation of meat [39]. Thus, TVB-N levels are essential indicators of the freshness of meat products. Regardless of the sterilization method, the TVB-N concentration increased with increased storage time (*p* < 0.05). In the later stages of storage (10 d), a large number of metabolites accumulated after protein decomposition, leading to a significant increase in TVB-N values [40]. However, at each storage time point, no significant difference was observed in the TVB-N content between the two sterilized treatments.

Ozone and hydroxyl radicals produced during plasma treatment are widely recognized as key reactive species responsible for breaking double bonds in unsaturated fatty acids, thereby inducing lipid oxidation [41]. Malondialdehyde, a common intermediate of lipid oxidation, can be quantified by TBA analysis. The difference in TBA content between LTPS and HSS became more pronounced as storage time increased. By the 10th day, the TBA levels in HSS samples were significantly higher than those in LTPS samples. Elevated TBA content is indicative of increased lipid oxidation, which contributes to quality degradation and the development of undesirable aroma production in meat products [42].

### 3.4. Protein Oxidation

Table 4 presents the degree of protein oxidation in LTPS- and HSS-treated samples during storage. Carbonyl content and sulfhydryl content are key indicators of protein oxidation. Sulfhydryl content is inversely related to protein oxidation whereas a lower carbonyl content indicates a lower degree of protein oxidation [43]. Regardless of the sterilization method chosen, the sulfhydryl content increased significantly, while the carbonyl content decreased significantly (*p* < 0.05), indicating that the protein in the beef brisket oxidized as storage time increased. Additionally, the thiol content in the HSS sample was significantly lower than that in the LTPS sample on the 10th day (*p* < 0.05), possibly due to the temperature treatment promoting the oxidation of sulfhydryl groups into disulfide bonds, thereby reducing the protein’s thiol content [44]. These results were also consistent with those observed in the carbonyl group. Previous studies have indicated that protein oxidation in meat products is a complex process and closely associated with fat oxidation, with both processes reinforcing each other. Aldehydes, ketones, and alcohols generated from the oxidation of unsaturated fatty acids in pork have been found to interact covalently/non-covalently with specific amino acid residues in proteins, thereby facilitating protein degradation [45]. This process could occur through two potential mechanisms: (1) free radicals or hydroperoxides produced during lipid oxidation attack sensitive amino acid side chains of proteins, initiating or accelerating protein oxidation; or (2) lipid oxidation products directly react with proteins, mediating protein oxidation [46].

### 3.5. Composition of Volatile Components

**Figure 3:**
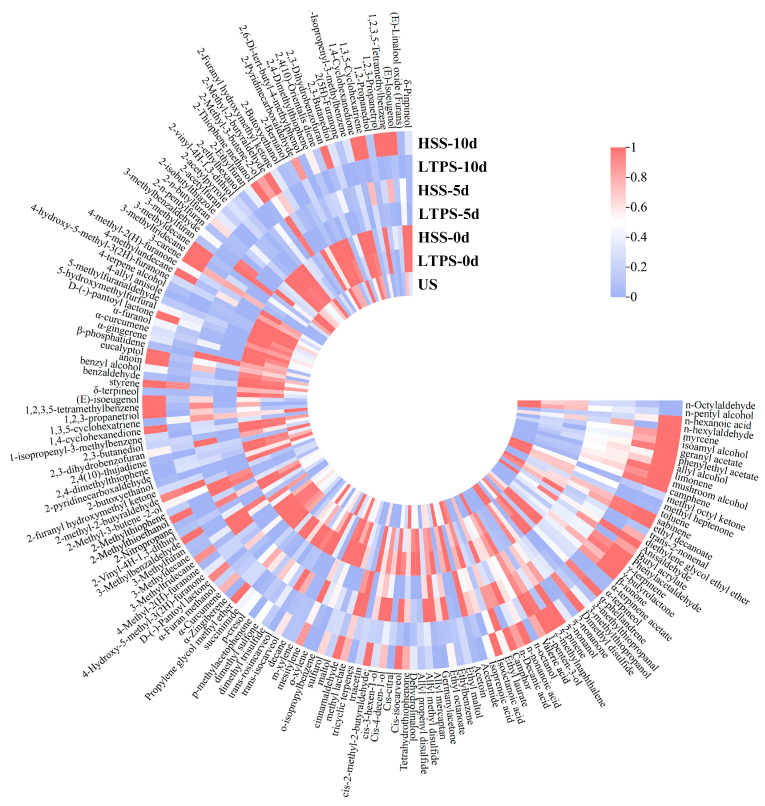
Heat map of volatile components in samples subject to different sterilization methods.

Figure 3 shows heatmaps of the volatile compounds in beef brisket during the 10-day storage period after different sterilization procedures. A total of 165 compounds were identified. Overall, the contents of volatile compounds decreased as storage time increased, indicating dissipation of the aroma. Additionally, there were greater numbers of volatile compound types in the HSS-treated samples compared with the LTPS-treated samples.

With an increase in storage time, the content of alcohol, ketone, and aldehyde compounds was significantly higher in the LTPS samples than in the HSS samples. These compounds, including nonanal and benzaldehyde, contribute to the characteristic aroma of meat. This phenomenon could be attributed to the conversion of lipid oxidation products, such as aldehydes and ketones, into flavor compounds [47]. Similar findings have been reported in previous studies [36,37,48,49], indicating appropriately intense low-temperature plasma treatment can enhance the flavor of meat. However, by the end of storage (10 d), the levels of sulfides and certain lipid oxidation products increased, contributing to the development of undesirable aromas [50,51]. Compounds such as dimethyl disulfide, 3-methylthiopropanol, valeraldehyde, and 2,4-decadienal were among those responsible for these negative sensory changes. Moreover, as natural flavor carriers, terpenoid compounds typically exhibit floral and woody aroma profiles, and their thermolabile characteristics are particularly critical in process selection [52]. For instance, the myrcene and eucalyptol contents in the LTPS-treated group were significantly higher than those in the HSS group. This phenomenon may be attributed to thermal sterilization-induced cleavage of monoterpene C=C bonds, whereas reactive nitrogen species primarily induced lipid peroxidation of microbial membranes within the effective treatment duration without disrupting the conjugated diene systems of terpenoids, thereby preserving the integrity of their aroma [53,54].

Furthermore, the VIP scores of the samples were calculated using orthogonal partial least squares-discriminant analysis (OPLS-DA). Typically, the key aroma differentiators among samples are compounds with OAVs > 1 and VIP > 1. As shown in Table 5, 37 such compounds were identified and ranked in descending order based on their OAVs. Benzaldehyde, decanoic acid, ethyl ester, 2-decanone, trans-β-ionone, and geranyl acetate were the five most influential compounds contributing to the aroma, each with OAVs > 100. Additionally, the OAVs gradually decreased with increased storage time. These findings highlight the role of these compounds in shaping the aroma profile of beef brisket at different stages of storage.

### 3.6. Electronic Nose

**Figure 4:**
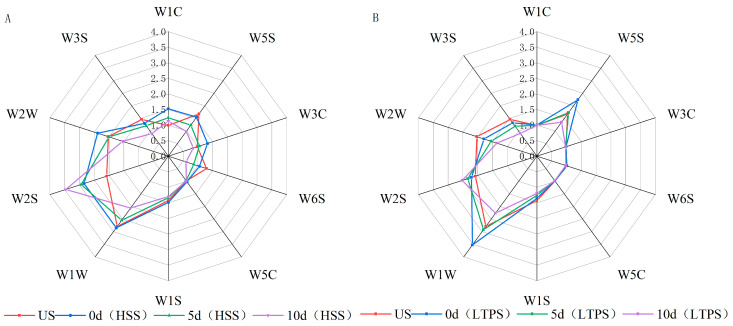
Radar plot of E-nose for HSS-treated samples (**A**) and LTPS-treated samples (**B**).

Figure 4A,B illustrate the electronic nose (E-nose) response values for tomato-braised beef brisket subjected to different sterilization treatments over a 10-day storage period. The E-nose sensor responses indicate dynamic changes in the concentration of volatile odor compounds within the sample headspace. Even minor variations in the concentration of volatile compounds are sufficient to produce detectable changes in sensor signals [55]. Notably, sensors W5S, W1W, and W2S exhibited significantly higher response values, corresponding to nitrogen oxides, alcohols, aldehydes, ethers, ketones, and sulfur-containing compounds. During storage, a general decline in response values was observed across most sensors, suggesting a gradual dissipation of aromatic compounds over time. The higher response of the W1W sensor indicated an accumulation of lipid oxidation products, such as aldehydes and ketones, during storage. This finding was consistent with the analysis of volatile components using GC-MS, which identified these compounds as primary oxidation byproducts. Compared to HSS treatment, LTPS treatment induced smaller fluctuations in e-nose response values, indicating better retention of the original aroma profile in LTPS-treated samples. Under high-voltage plasma discharge, higher NOx levels were detected in LTPS-treated samples likely due to the ionization of atmospheric oxygen and nitrogen. This ionization led to the generation of ROS and RONS, such as ozone (O₃) and nitric oxide (NO) [31]. The strong response of the W2S sensor (sulfur compounds) could be attributed to the following two factors: first, the beef brisket contained sulfur-containing volatiles, which contributed to the baseline signal [51]. Second, the emergence of undesirable sulfur-like odors during later storage stages likely originated from protein degradation or microbial degradation. These off-flavors, often linked to spoilage, are associated with compounds, such as hydrogen sulfide (H_2_S) or methanethiol [56].

**Figure 5:**
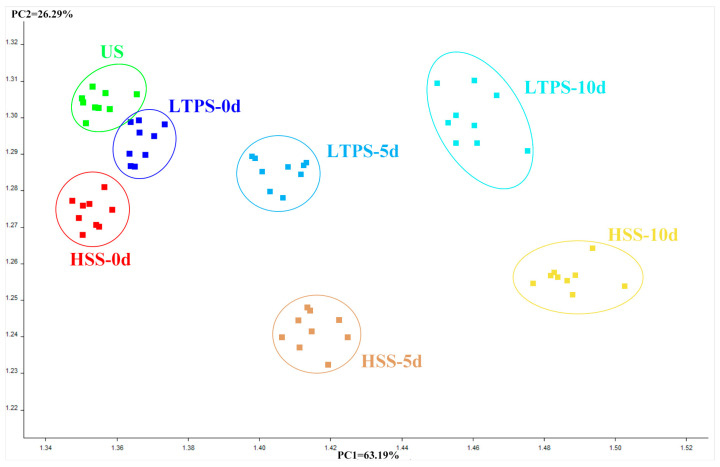
PCA plot of E-nose.

A principal component analysis (PCA) biplot was constructed to intuitively interpret the relationship between different types of samples and e-nose sensors. As illustrated in Figure 5, PC1 and PC2 accounted for 63.19% and 26.29% variance, respectively. On day 0, US, LTPS, and HSS samples clustered closely together, with US and HSS exhibiting the highest similarity. However, with an increase in storage time, the distance between HSS and US samples on the PC1 axis and PC2 axis gradually increased, reflecting growing differences in volatile flavor profiles. This observation suggested that compared to US controls, HSS-treated samples experienced more pronounced changes in volatile compounds during storage. In contrast, LTPS samples exhibited minimal shifts in PCA space over time, highlighting their superior stability in preserving aroma. This result aligned with the hypothesis that LTPS mitigates oxidative degradation and microbial activity, thereby maintaining the original volatile composition of tomato-braised beef brisket more effectively than HSS. The observed clustering patterns further support the conclusion that LTPS is a promising sterilization method for preserving the sensory quality in ready-to-eat meat products during extended storage.

### 3.7. Principal Component Analysis

**Figure 6:**
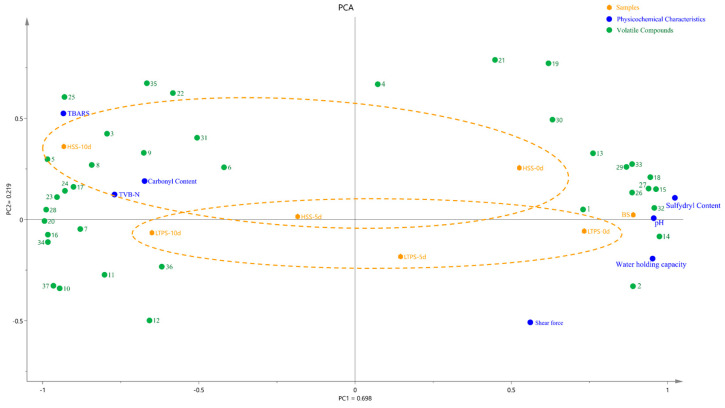
PCA biplot illustrating samples, physicochemical characteristics, and volatile components during storage. Note: the volatile components in Figure 6 are the primary contributors to aroma, as identified in Table S1. These compounds are listed as follows: 1. octanal; 2. 1-pentanol; 3. hexanoic acid; 4. hexanal; 5. β-Myrcene; 6. 1-butanol, 3-methyl-; 7. Geranyl acetate; 8. Acetic acid, 2-phenylethyl ester; 9. 2(2-Ethoxyethoxy)ethanol; 10. 2-Propen-1-ol; 11. pentanoic acid; 12. limonene; 13. 1-octen-3-ol; 14. camphene; 15. 2-decanone; 16. 2-methyl-hept-2-ene-6-one; 17. toluene; 18. sabinene; 19. decanoic acid, ethyl ester; 20. 2-nonenal, (E)-; 21. disulfide, dimethyl; 22. benzaldehyde; 23. 2-propenoic acid, butyl ester; 24. hyacinthin; 25. γ-terpinene; 26. butyrolactone; 27. trans-β-ionone; 28. α-terpinyl acetate; 29. α-terpineol; 30. α-phellandrene; 31. methional; 32. 3-methyl thiopropaol; 33. 2-nonanone; 34. 2-nonanol; 35. α-pinene; 36. naphthalene, 2-methyl-; 37. 1-penten-3-ol.

Figure 6 illustrates a PCA biplot that integrates physicochemical properties, protein oxidation levels, and key differential flavor compounds. The first two principal components, PC1 and PC2, explain 69.8% and 21.9% of the variance, respectively, resulting in a cumulative variance of 85.4%. The HSS samples at later storage stages (day 10) were positioned along the positive half-axis of PC2, whereas LTPS samples clustered along the negative half-axis. During the initial storage (day 0), LTPS were more closely aligned to the US samples than HSS samples. Additionally, the minimal spatial shifts observed in LTPS samples over time suggested that LTPS had a less pronounced impact on the physicochemical and oxidative stability compared to HSS (Yellow dot lines in Figure 6). Regardless of the sterilization method, more volatile components with positive aroma values were found in samples at the initial-storage time point. These compounds included 2-Nonenal, (E)- and octanal, characterized by beef and green aromas, respectively. In contrast, volatile compounds at the later storage time point (10 d) were dominated by aldehydes, ketones and sulfides, with sulfur-like and spicyaromas, including methional, hyacinthin and 2(2-Ethoxyethoxy) ethanol. This spatial pattern was consistent with the PCA results from the E-nose analysis (Figure 5), further supporting the consistency between aroma and physicochemical degradation trends.

Furthermore, increased TBARS, TVB-N, and carbonyl content were detected in both HSS- and LTPS-treated samples at the end of storage (10 days), suggesting advanced lipid and protein oxidation. The close clustering of these samples suggests a potential synergistic interaction between protein and lipid oxidation pathways [41], which could contribute to accelerated quality deterioration in pre-prepared dishes. This interplay between oxidative mechanisms warrants further investigation to better understand its impact on sensory and nutritional degradation.

## 4. Conclusions

This study systematically investigated the effects of LTPS on the quality of pre-prepared tomato-braised beef brisket with potatoes during storage. Freshness indicators TBARS and TVB-N were measured to evaluate the dynamics of protein and lipid oxidation. Furthermore, the carbonyl and sulfhydryl content of the samples were also measured during the storage period to assess the extent of protein oxidation. Volatile compounds were identified via GC-MS, and OAVs were utilized to determine the key aroma contributors. The results indicated a rise in oxidative markers (TBARS and carbonyl content) and a decline in freshness indicators (sulfhydryl content and water-holding capacity) as storage time increased irrespective of the sterilization method used.

LTPS treatment effectively suppressed microbial growth and mitigated oxidative damage, leading to lower lipid oxidation and better preservation of protein integrity compared to HSS-treated samples. HS-SPME-GCMS identified a total of 165 volatile compounds. By using orthogonal partial least-squares-discriminant analysis (OPLS-DA), the five most critical differentiating flavor compounds—benzaldehyde, decanoic acid, ethyl ester, 2-decanone, trans-β-ionone, and geranyl acetate—were identified, all with OAVs > 100. The OAVs of these five compounds in the LTPS-treated samples were more similar to those of the US samples, and differed markedly from those of the HSS-treated samples. Additionally, LTPS effectively preserved aldehydes and ketones associated with meaty aromas, whereas HSS-treated samples exhibited elevated sulfur-like off-flavors and oxidized lipid derivatives. E-nose and PCA analyses further confirmed the aroma stability of LTPS-treated samples, which clustered tightly, whereas HSS-treated samples diverged significantly due to volatile degradation.

Overall, the results of this study indicate that LTPS effectively minimized oxidative damage and flavor loss. This study provides a theoretical basis for optimizing LTPS parameters in industrial applications. Its non-thermal nature and compatibility with flexible packaging further support its suitability for sustainable preservation technology. While LTPS has been explored in limited food industry applications, its potential warrants further investigation. Future research in this area should focus on additional factors influencing LTPS technology, such as the role of carrier gas.

## Figures and Tables

**Table 1:** Performance of the electronic nose sensor array.

Number	Sensor Code	Performance
R1	W1C	Sensitive to aromatic compounds
R2	W5S	Sensitive to nitrogen oxides
R3	W3C	Sensitive to ammonia and aromatic compounds
R4	W6S	Sensitive to hydrogen compounds
R5	W5C	Sensitive to alkanes and aromatic compounds
R6	W1S	Sensitive to short-chain alkanes
R7	W1W	Sensitive to alcohols, aldehydes, ethers, and ketones
R8	W2S	Sensitive to inorganic sulfur and terpene compounds
R9	W2W	Sensitive to organic sulfur and terpene compounds
R10	W3S	Sensitive to long chain alkanes

**Table 2:** Effects of various sterilization treatments (carrier gas, voltage, and sterilization time) on the total number of colonies.

Different Sterilization Treatments (Carrier Gas, Voltage, and Sterilization Time)	Total Viable Count		
	24 h (lg(CFU/g))	48 h (lg(CFU/g))	72 h (lg(CFU/g))
US	0.95 ± 0.05 ^a^	2.51 ± 0.4 ^a^	4.47 ± 0.08 ^a^
HSS	0.53 ± 0.21 ^c^	1.15 ± 0.06 ^fg^	1.17 ± 0.08 ^f^
Air 120 KV 150 S	0.9 ± 0.05 ^a^	1.9 ± 0.28 ^b^	2.88 ± 0.18 ^b^
Air 120 KV 300 S	0.88 ± 0.09 ^a^	1.81 ± 0.25 ^b^	2.51 ± 0.02 ^c^
Air 120 KV 450 S	0.92 ± 0.06 ^a^	1.69 ± 0.22 ^bcd^	2.37 ± 0.11 ^c^
Air 140 KV 150 S	0.8 ± 0.08 ^abc^	1.74 ± 0.24 ^bc^	2.82 ± 0.06 ^b^
Air 140 KV 300 S	0.84 ± 0.06 ^ab^	1.57 ± 0.19 ^cde^	2.37 ± 0.03 ^c^
Air 140 KV 450 S	0.84 ± 0.06 ^ab^	1.37 ± 0.13 ^ef^	2.26 ± 0.04 ^c^
Air 160 KV 150 S	0.67 ± 0.06 ^abc^	1.5 ± 0.17 ^de^	1.94 ± 0.08 ^d^
Air 160 KV 300 S	0.68 ± 0.14 ^abc^	1.35 ± 0.13 ^ef^	1.95 ± 0.12 ^d^
Air 160 KV 450 S	0.55 ± 0.13 ^bc^	1.1 ± 0.04 ^g^	1.66 ± 0.07 ^e^

Note: different letters denote statistically significant differences (*p* < 0.05) between treatments.

**Table 3:** Effects of different sterilization methods on the physicochemical properties of tomato-stewed beef brisket during storage.

Storage Time(d)	Shear Force/gf		pH		WHC/%	
	LTPS	HSS	LTPS	HSS	LTPS	HSS
US	2401.59 ± 187.70 ^ab^	2422.08 ± 176.38 ^ab^	7.2 ± 0.41 ^a^	7.51 ± 0.32 ^a^	28.76 ± 0.14 ^a^	29.04 ± 0.12 ^a^
0	2414.33 ± 143.46 ^ab^	2491.64 ± 253.11 ^ab^	6.79 ± 0.32 ^a^	6.51 ± 0.60 ^a^	29.45 ± 0.31 ^a^	28.33 ± 0.16 ^a^
5	2362.79 ± 264.70 ^abc^	2046.94 ± 208.12 ^b^	6.51 ± 0.34 ^ab^	6.22 ± 0.74 ^a^	28.79 ± 0.32 ^b^	27.82 ± 0.13 ^b^
10	2268.63 ± 252.11 ^bcd^	1993.09 ± 232.24 ^ab^	6.09 ± 0.75 ^bc^	5.54 ± 0.46 ^ab^	28.42 ± 0.17 ^bc^	26.85 ± 0.06 ^c^

Note: different letters denote statistically significant differences (*p* < 0.05) between treatments.

**Table 4:** Changes in the degree of protein oxidation during storage.

Days	Sulfhydryl Content/nmol/mg		Carbonyl Content/nmol/mg	
	HSS	LTPS	HSS	LTPS
US	383.63 ± 9.48 ^a^	384.84 ± 5.04 ^a^	175.18 ± 10.32 ^d^	174.87 ± 8.32 ^cd^
0	335.75 ± 6.05 ^a^	354.37 ± 9.90 ^b^	198.11 ± 14.63 ^bc^	185.61 ± 7.60 ^bc^
5	312.87 ± 5.27 ^bc^	320.05 ± 7.68 ^b^	207.43 ± 23.95 ^b^	198.66 ± 14.44 ^bc^
10	272.61 ± 5.08 ^d^	302.00 ± 4.93 ^bc^	254.73 ± 6.66 ^a^	222.94 ± 6.56 ^b^

Note: different letters indicate statistically significant differences (*p* < 0.05) between treatments.

**Table 5:** Odor activity values of the key differential flavor compounds in samples after different types of sterilization.

CAS	Compound	Odor Description	*m*/*z*	OAVs						
				US	LTPS-0d	LTPS-5d	LTPS-10d	HSS-0d	HSS-5d	HSS-10d
123-11-5	Benzaldehyde	sweet, powdery, mimosa, floral, beef	135, 136, 77	1606.81	2457.74	3604.89	5260.61	246.13	360.67	527.96
110-38-3	Decanoic acid, ethyl ester	sweet, waxy, fruity, apple, grape	97, 102	925.57	1051.68	1207.77	1423.40	5.05	3.04	1.85
79-77-6	trans-β-Ionone	dry, powdery, floral, woody, orris	177, 43, 91	249.55	409.87	503.69	802.03	3.06	3.71	6.20
693-54-9	2-Decanone	orange, floral, fatty, peach	58, 43, 71	951.02	535.74	100.07	208.57	190.84	167.30	111.82
105-87-3	Geranyl acetate	floral, rose, lavender, green, waxy	69, 41, 43	68.03	91.00	122.54	176.94	13.93	19.26	27.23
91-57-6	Naphthalene, 2-methyl-	sweet, floral, woody	142, 141, 115	115.37	141.34	161.74	177.03	0.48	1.01	2.28
80-56-8	α-Pinene	fresh, camphor, sweet, pine, earthy	93, 91, 39	23.23	27.84	98.58	132.67	59.74	209.35	281.63
3387-41-5	sabinene	woody, terpene, citrus, pine, spice	93, 91, 77	31.98	48.88	76.19	90.87	6.25	6.72	7.20
142-62-1	Hexanoic acid	sour, fatty, sweat, cheese	60, 73, 41	37.77	48.27	51.76	81.10	35.00	40.27	80.99
123-35-3	β-Myrcene	peppery, terpene, spicy, balsam, plastic	41, 69, 93	20.55	27.43	35.77	47.11	31.10	44.51	62.22
616-25-1	1-Penten-3-ol	ethereal, horseradish, green, radish	57, 29, 27	14.23	16.49	33.99	40.91	6.77	14.49	16.66
108-88-3	Toluene	sweet	91, 92, 65	116.14	87.78	43.10	26.92	48.19	75.47	91.04
109-52-4	Pentanoic acid	sickening, putrid, acidic, sweaty, rancid	60, 73, 41	3.46	6.67	14.59	22.56	169.94	221.67	273.68
96-48-0	Butyrolactone	creamy, oily, fatty, caramel	42, 28, 41	17.64	19.90	21.79	22.47	20.32	22.69	24.17
3391-86-4	1-Octen-3-ol	mushroom, earthy, green, oily, fungal	57, 43, 72	6.71	8.06	16.67	21.47	99.03	137.06	179.11
821-55-6	2-Nonanone	fresh, sweet, green, weedy	43, 58, 41	135.43	87.16	58.81	20.19	8.74	6.34	2.46
628-99-9	2-Nonanol	waxy, creamy, citrus, orange, cheese	45, 69, 55	47.13	43.83	34.68	19.87	2.62	2.47	1.71
99-85-4	γ-Terpinene	oily, woody, terpene, lemon/lime	93, 91, 136	4.58	6.21	10.26	18.93	6.71	10.66	19.40
138-86-3	Limonene	citrus, herbal, terpene, camphor	68, 93, 67	10.66	11.54	15.07	18.50	8.15	17.40	23.33
98-55-5	α-Terpineol	pine, terpene, lilac, citrus, woody, floral	59, 93, 121	34.63	39.65	21.97	15.02	119.67	66.15	47.03
505-10-2	3-Methyl thiopropaol	sulfurous, onion, sweet, soup, vegetable	106, 61, 58	23.77	32.35	9.70	13.90	4.32	1.46	2.48
123-51-3	1-Butanol, 3-methyl-	fusel, oil, alcoholic, whiskey, fruity	55, 42, 43	7.63	7.92	9.82	13.34	7.15	12.35	20.05
71-41-0	1-Pentanol	fusel, oil, sweet, balsam	42, 55, 41	17.67	16.26	14.08	12.24	47.06	39.11	31.28
103-45-7	Acetic acid, 2-phenylethyl ester	floral, rose, sweet, honey, fruity	104, 43, 91	4.02	5.27	9.91	10.11	1.63	2.54	3.55
80-26-2	α-Terpinyl acetate	herbal, bergamot, lavender, lime, citrus	43, 121, 93	25.46	35.87	17.59	9.42	89.87	44.05	24.86
18829-56-6	2-Nonenal, (E)-	fatty, green, cucumber, aldehydic, citrus	43, 55, 70	36.98	25.82	11.87	7.20	2.65	1.63	2.26
624-92-0	Disulfide, dimethyl	sulfurous, vegetable, cabbage, onion	93, 79, 45	1.58	1.39	2.79	6.09	1.86	3.38	7.06
79-92-5	Camphene	woody, herbal, fir, needle, camphor	93, 121, 79	3.24	3.29	4.56	5.91	4.52	0.96	2.48
66-25-1	Hexanal	fresh, green, fatty, aldehydic, grass, leafy, fruity, sweaty	44, 56, 41	7.16	5.99	4.93	3.45	354.28	390.18	450.87
122-78-1	Hyacinthin	green, sweet, floral, hyacinth, clover, honey, cocoa	91, 92, 120	1.66	2.35	2.40	3.41	15.57	15.52	22.70
107-18-6	2-Propen-1-ol	pungent, mustard	57, 31, 39	2.46	2.46	2.98	3.17	4.16	9.04	13.64
3268-49-3	Methional	musty, potato, earthy, vegetable	48, 104, 47	10.06	10.57	1.63	2.14	53.38	8.60	12.17
124-13-0	Octanal	aldehydic, waxy, citrus, orange, peel, green, herbal, fresh, fatty	44, 45, 41	11.68	8.12	3.75	1.80	21.45	9.04	2.15
110-93-0	2-methyl-hept-2-ene-6-one	citrus, green, musty, lemongrass, apple	43, 41, 69	3.29	2.81	2.45	1.64	29.86	14.62	9.50
99-83-2	α-Phellandrene	citrus, herbal, terpene, green	93, 91, 77	0.36	0.49	0.76	1.21	20.56	30.85	49.95
111-90-0	2(2-Ethoxyethoxy)ethanol	slightly, ethereal	45, 59, 31	1.56	1.38	0.78	0.54	12.39	15.27	17.57
141-32-2	2-Propenoic acid, butyl ester	-	55, 56, 73	1.49	0.72	0.42	0.06	1.41	1.29	1.25

Note: Details of the key differential flavor compounds are presented in Supplementary Table S1, including the RI (retention index), reference RI, and threshold values.

## Data Availability

The original contributions presented in this study are included in the article/Supplementary Materials. Further inquiries can be directed to the corresponding authors.

## Supplementary Materials

The following supporting information can be downloaded at https://www.mdpi.com/article/10.3390/foods14071106/s1, Table S1: the key differential flavor compounds.

## References

[1] Convenience Food—Worldwide. Statista Market Forecast. https://www.statista.com/outlook/cmo/food/convenience-food/worldwide.

[2] Pérez-Rodríguez F., Zamorano A.R., Posada-Izquierdo G.D., García-Gimeno R.M. (2014). Study of the Effect of Post-Packaging Pasteurization and Argon Modified Atmosphere Packaging on the Sensory Quality and Growth of Endogenous Microflora of a Sliced Cooked Meat Product. Food Sci. Technol. Int..

[3] Capitain C., Weller P. (2021). Non-Targeted Screening Approaches for Profiling of Volatile Organic Compounds Based on Gas Chromatography-Ion Mobility Spectroscopy (GC-IMS) and Machine Learning. Molecules.

[4] Cui Z., Yan H., Manoli T., Mo H., Li H., Zhang H. (2020). Changes in the Volatile Components of Squid (*Illex argentinus*) for Different Cooking Methods via Headspace—Gas Chromatography–Ion Mobility Spectrometry. Food Sci. Nutr..

[5] Bi J., Ping C., Chen Z., Yang Z., Li B., Gao Y., Zhang Y., He H. (2024). Evaluating the Influence of High-Temperature Sterilization and Pasteurization on Volatile Organic Compounds in Tomato Stewed Beef Brisket: An Analysis Using Gas Chromatography-Ion Mobility Spectrometry and Multivariate Statistical Visualization. Int. J. Gastron. Food Sci..

[6] Zhao C., Dai J., Chen F., Zhao Z., Zhao X. (2023). The Effect of Different Sterilization Methods on the Shelf Life and Physicochemical Indicators of Fermented Pork Jerky. Front. Nutr..

[7] Li X., Wang L., Wu W. (2018). Exploration of Ultra-High Pressure Sterilization on Cooked Duck Meat. Storage Process.

[8] Sun M., Ran P., Huang Y. (2024). Effect of Ultra-High-Pressure Sterilization on Flavor and Physicochemical Properties of Low-Salt Sliced Bacon. Sci. Technol. Food Ind..

[9] Zhang J., Toldra F., Kang D., Zhou L., Wang J., Zhang W., Hu Y. (2024). Benefits of Ultrasonic Technology Application in Meat Field and Its Influential Mechanism: A Review. Crit. Rev. Food Sci. Nutr..

[10] Lee J., Lee C.W., Yong H.I., Lee H.J., Jo C., Jung S. (2017). Use of Atmospheric Pressure Cold Plasma for Meat Industry. Korean J. Food Sci. Anim. Resour..

[11] Jayasena D.D., Kang T., Wijayasekara K.N., Jo C. (2023). Innovative Application of Cold Plasma Technology in Meat and Its Products. Food Sci. Anim. Resour..

[12] Akhtar J., Abrha M.G., Teklehaimanot K., Gebrekirstos G. (2022). Cold Plasma Technology: Fundamentals and Effect on Quality of Meat and Its Products. Food Agric. Immunol..

[13] Barjasteh A., Kaushik N., Choi E.H., Kaushik N.K. (2024). Cold Atmospheric Pressure Plasma Solutions for Sustainable Food Packaging. Int. J. Mol. Sci..

[14] Kim B., Yun H., Jung S., Jung Y., Jung H., Choe W., Jo C. (2011). Effect of Atmospheric Pressure Plasma on Inactivation of Pathogens Inoculated onto Bacon Using Two Different Gas Compositions. Food Microbiol..

[15] Yuan H., Chen F., Zhang J., Guo X., Zhang J., Yan W. (2025). Investigating the Synergistic Bactericidal Effects of Cold Plasma and Ultraviolet Radiation on *Pseudomonas fragi*. Foods.

[16] Chen Y., He Y., Jin T., Dai C., Xu Q., Wu Z. (2023). Bactericidal Effect of Low-Temperature Atmospheric Plasma against the *Shigella flexneri*. Biomed. Eng. Online.

[17] Li P., Zhang H., Tian C., Zou H. (2024). Experimental Investigation of Bacterial Inactivation of Beef Using Indirect Cold Plasma in Cold Chain and at Room Temperature. Foods.

[18] Sriraksha M.S., Ayenampudi S.B., Noor M., Raghavendra S.N., Chakka A.K. (2023). Cold Plasma Technology: An Insight on Its Disinfection Efficiency of Various Food Systems. Food Sci. Technol. Int..

[19] Qian J., Ma L., Yan W., Zhuang H., Huang M., Zhang J., Wang J. (2022). Inactivation Kinetics and Cell Envelope Damages of Foodborne Pathogens Listeria Monocytogenes and Salmonella Enteritidis Treated with Cold Plasma. Food Microbiol..

[20] Luo J., Nasiru M.M., Yan W., Zhuang H., Zhou G., Zhang J. (2020). Effects of Dielectric Barrier Discharge Cold Plasma Treatment on the Structure and Binding Capacity of Aroma Compounds of Myofibrillar Proteins from Dry-Cured Bacon. LWT-Food Sci. Technol..

[21] Abdel-Naeem H.H.S., Ebaid E.M.S.M., Khalel K.H.M., Imre K., Morar A., Herman V., EL-Nawawi F.A.M. (2022). Decontamination of Chicken Meat Using Dielectric Barrier Discharge Cold Plasma Technology: The Effect on Microbial Quality, Physicochemical Properties, Topographical Structure, and Sensory Attributes. LWT-Food Sci. Technol..

[22] Zhang H., Zhang C., Han Q. (2023). Mechanisms of Bacterial Inhibition and Tolerance around Cold Atmospheric Plasma. Appl. Microbiol. Biotechnol..

[23] Qiang Y., Wang J., Jiang W., Wang T., Huang F., Han D., Zhang C. (2025). Insights into the Flavor Endowment of Aroma-Active Compounds in Cloves (*Syzygium aromaticum*) to Stewed Beef. Food Chem..

[24] Wang Y., Jiang Z., Qian J., Dai J. (2022). Effect of Cold Plasma on Microbial Decontamination and Storage Quality of Fish Fillets. Food Ferment. Ind..

[25] Gharibzahedi S.M.T., Mohammadnabi S. (2017). Effect of Novel Bioactive Edible Coatings Based on Jujube Gum and Nettle Oil-Loaded Nanoemulsions on the Shelf-Life of Beluga Sturgeon Fillets. Int. J. Biol. Macromol..

[26] Xiong Z., Sun D.-W., Pu H., Xie A., Han Z., Luo M. (2015). Non-destructive prediction of thiobarbituric acid reactive substances (TBARS) value for freshness evaluation of chicken meat using hyperspectral imaging. Food Chem..

[27] Bao Y., Ertbjerg P. (2015). Relationship between Oxygen Concentration, Shear Force and Protein Oxidation in Modified Atmosphere Packaged Pork. Meat Sci..

[28] Shi Q., Xiao Y., Zhou Y., Tang W. (2024). Comparison of Ultra-High-Pressure and Conventional Cold Brew Coffee at Different Roasting Degrees: Physicochemical Characteristics and Volatile and Non-Volatile Components. Foods.

[29] Niu Y., Wang R., Xiao Z., Zhu J., Sun X., Wang P. (2019). Characterization of Ester Odorants of Apple Juice by Gas Chromatography-Olfactometry, Quantitative Measurements, Odour Threshold, Aroma Intensity and Electronic Nose. Food Res. Int..

[30] (2022). Determination of the Total Number of Colonies in Food Microbiological Inspection of China Food Safety National Standard.

[31] (2005). Commission Regulation (EC) No 2073/2005 of 15 November 2005 on Microbiological Criteria for Foodstuffs.

[32] Harikrishna S., Anil P.P., Shams R., Dash K.K. (2023). Cold Plasma as an Emerging Nonthermal Technology for Food Processing: A Comprehensive Review. J. Agric. Food Res..

[33] Feizollahi E., Misra N.N., Roopesh M.S. (2021). Factors Influencing the Antimicrobial Efficacy of Dielectric Barrier Discharge (DBD) Atmospheric Cold Plasma (ACP) in Food Processing Applications. Crit. Rev. Food Sci. Nutr..

[34] Kim H.-J., Yong H.I., Park S., Choe W., Jo C. (2013). Effects of Dielectric Barrier Discharge Plasma on Pathogen Inactivation and the Physicochemical and Sensory Characteristics of Pork Loin. Curr. Appl. Phys..

[35] Misra N.N., Jo C. (2017). Applications of Cold Plasma Technology for Microbiological Safety in Meat Industry. Trends Food Sci. Technol..

[36] Wang C. (2021). Effects of Cold Plasma Sterilization on Shelf Life and Flavor Quality of Salted Duck. Sci. Technol. Food Ind..

[37] Bauer A., Ni Y., Bauer S., Paulsen P., Modic M., Walsh J.L., Smulders F.J.M. (2017). The Effects of Atmospheric Pressure Cold Plasma Treatment on Microbiological, Physical-Chemical and Sensory Characteristics of Vacuum Packaged Beef Loin. Meat Sci..

[38] Qiu Y. (2024). Overview on Changes and Mechanism of Low-Temperature Plasma Sterilization on Edible Quality of Meat Products. Food Ferment. Ind..

[39] Jung E.-Y., Yun I.-R., Go G., Kim G.-D., Seo H.-W., Joo S.-T., Yang H.-S. (2012). Effects of *Radix puerariae* Extracts on Physicochemical and Sensory Quality of Precooked Pork Sausage during Cold Storage. LWT-Food Sci. Technol..

[40] Dong Y., Wang H., Xu X. (2012). Storage Characteristics and Shelf Life Prediction of Vacuum-Packed Salted Goose under Different Storage Temperatures. Food Sci..

[41] Sarangapani C., Keogh D.R., Dunne J., Bourke P., Cullen P.J. (2017). Characterisation of Cold Plasma Treated Beef and Dairy Lipids Using Spectroscopic and Chromatographic Methods. Food Chem..

[42] Ding J., Hu X., Lu X. (2017). Storage Characteristics and Shelf-Life Prediction of Spicy Beef under Different Storage Temperatures. Mod. Food Sci. Technol..

[43] Nawaz A., Irshad S., Khan I.A., Khalifa I., Walayat N., Aadil R.M., Kumar M., Wang M., Chen F., Cheng K.-W. (2022). Protein Oxidation in Muscle-Based Products: Effects on Physicochemical Properties, Quality Concerns, and Challenges to Food Industry. Food Res. Int..

[44] Cheng J.-H., Chen Y.-Q., Sun D.-W. (2021). Effects of Plasma Activated Solution on the Colour and Structure of Metmyoglobin and Oxymyoglobin. Food Chem..

[45] Segat A., Misra N.N., Cullen P.J., Innocente N. (2015). Atmospheric Pressure Cold Plasma (ACP) Treatment of Whey Protein Isolate Model Solution. Innov. Food Sci. Emerg. Technol..

[46] Takai E., Kitano K., Kuwabara J., Shiraki K. (2012). Protein Inactivation by Low-Temperature Atmospheric Pressure Plasma in Aqueous Solution. Plasma Process. Polym..

[47] Li H., Chen J., Zhang Y., Jiang Y., Sun D., Piao C., Li T., Wang J., Li H., Mu B. (2024). Evaluation of the Flavor Profiles of Yanbian-Style Sauced Beef from Differently Treated Raw Beef Samples. Food Chem. X.

[48] Perez-Andres J.M., de Alba M., Harrison S.M., Brunton N.P., Cullen P.J., Tiwari B.K. (2020). Effects of Cold Atmospheric Plasma on Mackerel Lipid and Protein Oxidation during Storage. LWT-Food Sci. Technol..

[49] Nasiru M.M., Frimpong E.B., Muhammad U., Qian J., Mustapha A.T., Yan W., Zhuang H., Zhang J. (2021). Dielectric Barrier Discharge Cold Atmospheric Plasma: Influence of Processing Parameters on Microbial Inactivation in Meat and Meat Products. Compr. Rev. Food Sci. Food Saf..

[50] Huang M., Wang J., Zhuang H., Yan W., Zhao J., Zhang J. (2019). Effect of In-Package High Voltage Dielectric Barrier Discharge on Microbiological, Color and Oxidation Properties of Pork in Modified Atmosphere Packaging during Storage. Meat Sci..

[51] Wang X., Zhu L., Han Y., Xu L., Jin J., Cai Y., Wang H. (2018). Analysis of Volatile Compounds between Raw and Cooked Beef by HS-SPME–GC–MS. J. Food Process. Preserv..

[52] Wang T., Yang P., Huang F., Qiang Y., Han D. (2023). Interaction Mechanisms for the Muscle Proteins with Terpenoid Compounds during Heat Treatment. Trans. Chin. Soc. Agric. Eng..

[53] Pan Q., Shao X., Xiao Q., Gu Q., Chen C., Xu B., Li P. (2025). Revealing the Flavor Changes of Spiced Beef under Different Thermal Treatment Temperatures: A Complementary Approach with GC-IMS and GC-O-MS. Food Chem..

[54] Wang Q., Du R., Wang Y., Zhang S., Wang L., Wang L. (2024). Characterization and Differentiation of Flavor Profile of 12 Air-Dried Yak Meat Products Using GC-IMS and Multivariate Analysis. Foods.

[55] Shi C., Yang X., Han S., Fan B., Zhao Z., Wu X., Qian J. (2018). Nondestructive Prediction of Tilapia Fillet Freshness During Storage at Different Temperatures by Integrating an Electronic Nose and Tongue with Radial Basis Function Neural Networks. Food Bioprocess Technol..

[56] Lee H.J., Kwon J.A., Kim M., Lee Y.E., Ryu M., Jo C. (2023). Effect of Supercooling on Storage Ability of Different Beef Cuts in Comparison to Traditional Storage Methods. Meat Sci..

