# Supplementary material for: Effect of Low-Temperature Plasma Sterilization on the Quality of Pre-Prepared Tomato-Stewed Beef Brisket During Storage: Microorganism, Freshness, Protein Oxidation and Flavor Characteristics
Source: Foods. 2025 Mar 22;14(7):1106. doi: 10.3390/foods14071106 (PMC11988737; doi:10.3390/foods14071106)
Supplement: Supplementary file 1 [file foods-14-01106-s001.zip › foods-3524370-supplementary.pdf]

# Supplementary Material

# Effect of Low-Temperature Plasma Sterilization on the Quality of Pre-Prepared Tomato-Stewed Beef Brisket During Storage: Microorganism, Freshness, Protein Oxidation and Flavor Characteristics

**Qihan Shi [1], Ying Xiao [2,*], Yiming Zhou [1,*], Jinhong Wu [3], Xiaoli Zhou [1], Yanping Chen [3] and Xiaodan Liu [2]**

[1] School of Perfume and Aroma Technology, Shanghai Institute of Technology, Shanghai 201418, China; 13916902288@163.com (Q.S.); zhouxlsit@163.com (X.Z.)
[2] School of Food and Tourism, Shanghai Urban Construction Vocational College, Shanghai 201415, China; liuxiaodan@succ.edu.cn
[3] School of Agriculture and Biology, Shanghai Jiao Tong University, Shanghai 200240, China; wujinhong@sjtu.edu.cn (J.W.); catherinechenyp@163.com (Y.C.)
* Correspondence: xiaoying@succ.edu.cn (Y.X.); zhouymsit@163.com (Y.Z.)

## Content

**Table S1** Details of the key differential flavor compounds

| CAS | Compound | Odor、description | m/z | RI | Reference RI | Threshold | OAVs | | | | | | |
|---|---|---|---|---|---|---|---|---|---|---|---|---|---|
| | | | | | | | BS | LTPS-0d | LTPS-5d | LTPS-10d | HSS-0d | HSS-5d | HSS-10d |
| 123-11-5 | **Benzaldehyde** | sweet、powdery、mimosa、floral、hawthorn | 135、136、77 | 2019 | 2011 | 0.1 | 1606.81 | 2457.74 | 3604.89 | 5260.61 | 246.13 | 360.67 | 527.96 |
| 110-38-3 | **Decanoic acid, ethyl ester** | sweet、waxy、fruity、apple、grape | 97、102 | 1640 | 1638 | 0.005 | 925.57 | 1051.68 | 1207.77 | 1423.4 | 5.05 | 3.04 | 1.85 |
| 79-77-6 | **trans-β-Ionone** | dry、powdery、floral、woody、orris | 177、43、91 | 1938 | 1967 | 0.007 | 249.55 | 409.87 | 503.69 | 802.03 | 3.06 | 3.71 | 6.2 |
| 693-54-9 | **2-Decanone** | orange、floral、fatty、peach | 58、43、71 | 1492 | 1493 | 0.0083 | 951.02 | 535.74 | 100.07 | 208.57 | 190.84 | 167.3 | 111.82 |
| 105-87-3 | **Geranyl acetate** | floral、rose、lavender、green、waxy | 69、41、43 | 1756 | 1752 | 0.15 | 68.03 | 91 | 122.54 | 176.94 | 13.93 | 19.26 | 27.23 |
| 91-57-6 | **Naphthalene, 2-methyl-** | sweet、floral、woody | 142、141、115 | 1845 | 1856 | 0.003 | 115.37 | 141.34 | 161.74 | 177.03 | 0.48 | 1.01 | 2.28 |
| 80-56-8 | **α-Pinene** | fresh、camphor、sweet、pine、earthy | 93、91、39 | 1009 | 1027 | 2.12 | 23.23 | 27.84 | 98.58 | 132.67 | 59.74 | 209.35 | 281.63 |
| 3387-41-5 | **sabinene** | woody、terpene、citrus、pine、spice | 93、91、77 | 1104 | 1124 | 0.98 | 31.98 | 48.88 | 76.19 | 90.87 | 6.25 | 6.72 | 7.2 |
| 142-62-1 | **Hexanoic acid** | sour、fatty、sweat、cheese | 60、73、41 | 1848 | 1846 | 0.7 | 37.77 | 48.27 | 51.76 | 81.1 | 35 | 40.27 | 80.99 |
| 123-35-3 | **β-Myrcene** | peppery、terpene、spicy、balsam、plastic | 41、69、93 | 1153 | 1161 | 1.2 | 20.55 | 27.43 | 35.77 | 47.11 | 31.1 | 44.51 | 62.22 |
| 616-25-1 | **1-Penten-3-ol** | ethereal、horseradish、green、radish | 57、29、27 | 1162 | 1158 | 0.4 | 14.23 | 16.49 | 33.99 | 40.91 | 6.77 | 14.49 | 16.66 |

| | | | | | | | | | | | | | |
|---|---|---|---|---|---|---|---|---|---|---|---|---|---|
| 108-88-3 | **Toluene** | sweet | 91、92、65 | 1027 | 1042 | 0.33 | 116.14 | 87.78 | 43.1 | 26.92 | 48.19 | 75.47 | 91.04 |
| 109-52-4 | **Pentanoic acid** | sickening、putrid、acidic、sweaty、rancid | 60、73、41 | 1741 | 1733 | 0.6 | 3.46 | 6.67 | 14.59 | 22.56 | 169.94 | 221.67 | 273.68 |
| 96-48-0 | **Butyrolactone** | creamy、oily、fatty、caramel | 42、28、41 | 1619 | 1632 | 1 | 17.64 | 19.9 | 21.79 | 22.47 | 20.32 | 22.69 | 24.17 |
| 3391-86-4 | **1-Octen-3-ol** | mushroom、earthy、green、oily、fungal | 57、43、72 | 1452 | 1450 | 1 | 6.71 | 8.06 | 16.67 | 21.47 | 99.03 | 137.06 | 179.11 |
| 821-55-6 | **2-Nonanone** | fresh、sweet、green、weedy、earthyl | 43、58、41 | 1386 | 1390 | 0.1 | 135.43 | 87.16 | 58.81 | 20.19 | 8.74 | 6.34 | 2.46 |
| 628-99-9 | **2-Nonanol** | waxy、creamy、citrus、orange、cheese | 45、69、55 | 1523 | 1521 | 0.058 | 47.13 | 43.83 | 34.68 | 19.87 | 2.62 | 2.47 | 1.71 |
| 99-85-4 | **γ-Terpinene** | oily、woody、terpene、lemon/lime | 93、91、136 | 1233 | 1246 | 1 | 4.58 | 6.21 | 10.26 | 18.93 | 6.71 | 10.66 | 19.4 |
| 138-86-3 | **Limonene** | citrus、herbal、terpene、camphor | 68、93、67 | 1178 | 1199 | 14.7 | 10.66 | 11.54 | 15.07 | 18.5 | 8.15 | 17.4 | 23.33 |
| 98-55-5 | **α-Terpineol** | pine、terpene、lilac、citrus、woody、floral | 59、93、121 | 1698 | 1697 | 3 | 34.63 | 39.65 | 21.97 | 15.02 | 119.67 | 66.15 | 47.03 |
| 505-10-2 | **3-Methyl thiopropaol** | sulfurous、onion、sweet、soup、vegetable | 106、61、58 | 1715 | 1719 | 0.123 | 23.77 | 32.35 | 9.7 | 13.9 | 4.32 | 1.46 | 2.48 |
| 123-51-3 | **1-Butanol, 3-methyl-** | fusel、oil、alcoholic、whiskey、fruity | 55、42、43 | 1209 | 1209 | 1.296 | 7.63 | 7.92 | 9.82 | 13.34 | 7.15 | 12.35 | 20.05 |
| 71-41-0 | **1-Pentanol** | fusel、oil、sweet、balsam | 42、55、41 | 1253 | 1250 | 3 | 17.67 | 16.26 | 14.08 | 12.24 | 47.06 | 39.11 | 31.28 |
| 103-45-7 | **Acetic acid, 2-phenylethyl ester** | floral、rose、sweet、honey、fruity | 104、43、91 | 1813 | 1813 | 0.233 | 4.02 | 5.27 | 9.91 | 10.11 | 1.63 | 2.54 | 3.55 |

| | | | | | | | | | | | | | |
|---|---|---|---|---|---|---|---|---|---|---|---|---|---|
| 80-26-2 | **α-Terpinyl acetate** | herbal、bergamot、lavender、lime、citrus | 43、121、93 | 1695 | 1693 | 2.5 | 25.46 | 35.87 | 17.59 | 9.42 | 89.87 | 44.05 | 24.86 |
| 18829-56-6 | **2-Nonenal, (E)-** | fatty、green、cucumber、aldehydic、citrus | 43、55、70 | 1532 | 1534 | 0.19 | 36.98 | 25.82 | 11.87 | 7.2 | 2.65 | 1.63 | 2.26 |
| 624-92-0 | **Disulfide, dimethyl** | sulfurous、vegetable、cabbage、onion | 93、79、45 | 1060 | 1077 | 1.1 | 1.58 | 1.39 | 2.79 | 6.09 | 1.86 | 3.38 | 7.06 |
| 79-92-5 | **Camphene** | woody、herbal、fir、needle、camphor | 93、121、79 | 1051 | 1070 | 30 | 3.24 | 3.29 | 4.56 | 5.91 | 4.52 | 0.96 | 2.48 |
| 66-25-1 | **Hexanal** | fresh、green、fatty、aldehydic、grass、leafy、fruity、sweaty | 44、56、41 | 1071 | 1083 | 47 | 7.16 | 5.99 | 4.93 | 3.45 | 354.28 | 390.18 | 450.87 |
| 122-78-1 | **Hyacinthin** | green、sweet、floral、hyacinth、clover、honey、cocoa | 91、92、120 | 1634 | 1641 | 6.3 | 1.66 | 2.35 | 2.4 | 3.41 | 15.57 | 15.52 | 22.7 |
| 107-18-6 | **2-Propen-1-ol** | pungent、mustard | 57、31、39 | 1113 | 1123 | 5 | 2.46 | 2.46 | 2.98 | 3.17 | 4.16 | 9.04 | 13.64 |
| 3268-49-3 | **Methional** | musty、potato、earthy、vegetable | 48、104、47 | 1449 | 1454 | 5.0007005 | 10.06 | 10.57 | 1.63 | 2.14 | 53.38 | 8.6 | 12.17 |
| 124-13-0 | **Octanal** | aldehydic、waxy、citrus、orange、peel、green、herbal、fresh、fatty | 44、45、41 | 1284 | 1289 | 2.5077838 | 11.68 | 8.12 | 3.75 | 1.8 | 21.45 | 9.04 | 2.15 |
| 110-93-0 | **2-methyl-hept-2-ene-6-one** | citrus、green、musty、lemongrass、apple | 43、41、69 | 1334 | 1339 | 68 | 3.29 | 2.81 | 2.45 | 1.64 | 29.86 | 14.62 | 9.5 |

| | | | | | | | | | | | | | |
|---|---|---|---|---|---|---|---|---|---|---|---|---|---|
| 99-83-2 | **α-Phellandrene** | citrus、herbal、terpene、green | 93、91、77 | 1149 | 1167 | 40 | 0.36 | 0.49 | 0.76 | 1.21 | 20.56 | 30.85 | 49.95 |
| 111-90-0 | **2(2-Ethoxyethoxy)ethanol** | slightly、ethereal | 45、59、31 | 1620 | 1617 | 1.6 | 1.56 | 1.38 | 0.78 | 0.54 | 12.39 | 15.27 | 17.57 |
| 141-32-2 | **2-Propenoic acid, butyl ester** | - | 55、56、73 | 1171 | 1184 | 1 | 1.49 | 0.72 | 0.42 | 0.06 | 1.41 | 1.29 | 1.25 |
